# The archaeology of climate change: The case for cultural diversity

**DOI:** 10.1073/pnas.2108537118

**Published:** 2021-07-22

**Authors:** Ariane Burke, Matthew C. Peros, Colin D. Wren, Francesco S. R. Pausata, Julien Riel-Salvatore, Olivier Moine, Anne de Vernal, Masa Kageyama, Solène Boisard

**Affiliations:** ^a^Université de Montréal, Montreal, QC H3T 1J4, Canada;; ^b^Bishop’s University, Sherbrooke, QC J1M 1Z7, Canada;; ^c^University of Colorado, Colorado Springs, CO 80918;; ^d^Université de Québec à Montréal, Montreal, QC H2L 2C4, Canada;; ^e^UMR 8591 CNRS Université Paris 1, Université Paris-Est Créteil Val de Marne, 94010 Créteil, France;; ^f^Laboratoire des Sciences du Climat et de l’Environnement/Institut Pierre Simon Laplace, 91191 Gif-sur-Yvette, France

**Keywords:** archaeology, climate change, cultural diversity, resilience, climate science

## Abstract

Anthropogenic climate change is currently driving environmental transformation on a scale and at a pace that exceeds historical records. This represents an undeniably serious challenge to existing social, political, and economic systems. Humans have successfully faced similar challenges in the past, however. The archaeological record and Earth archives offer rare opportunities to observe the complex interaction between environmental and human systems under different climate regimes and at different spatial and temporal scales. The archaeology of climate change offers opportunities to identify the factors that promoted human resilience in the past and apply the knowledge gained to the present, contributing a much-needed, long-term perspective to climate research. One of the strengths of the archaeological record is the cultural diversity it encompasses, which offers alternatives to the solutions proposed from within the Western agro-industrial complex, which might not be viable cross-culturally. While contemporary climate discourse focuses on the importance of biodiversity, we highlight the importance of cultural diversity as a source of resilience.

Current efforts to curb global warming have been largely ineffective and future climate scenarios predict that global temperatures will rise from +2.6 to + 4.8 °C (and as much as +8 °C in the Arctic) by the end of the century (Fig. 1) (1–3). The scope of the ecological transformations that could occur beyond 2100 CE under prevailing emission rates is truly alarming (4). Planning a sustainable response to climate change requires us to identify the critical climate thresholds capable of disrupting social, economic, or political systems and culturally appropriate strategies for countering such disruptions. Natural climate archives (e.g., pollen data, sediment records, ice cores) and the paleontological and archaeological records offer unique opportunities for observing, measuring, and understanding how humans have responded to a wide range of climate events in the past, forming a sound basis for predicting how climate change could transform our lives in the future and offering a range of possible solutions (e.g., ref. 5). The archaeological record is a valuable source of information that has been largely overlooked in climate research until comparatively recently, however (6–12). As a result, the sensitivity of human systems to the full range of conditions predicted under different future climate scenarios remains largely untested. We contend that a multidisciplinary science of the past—an “archaeology of climate change”—provides a solid foundation for assessing the implications of climate change across cultures and helps design sustainable development strategies.

**Fig. 1.**
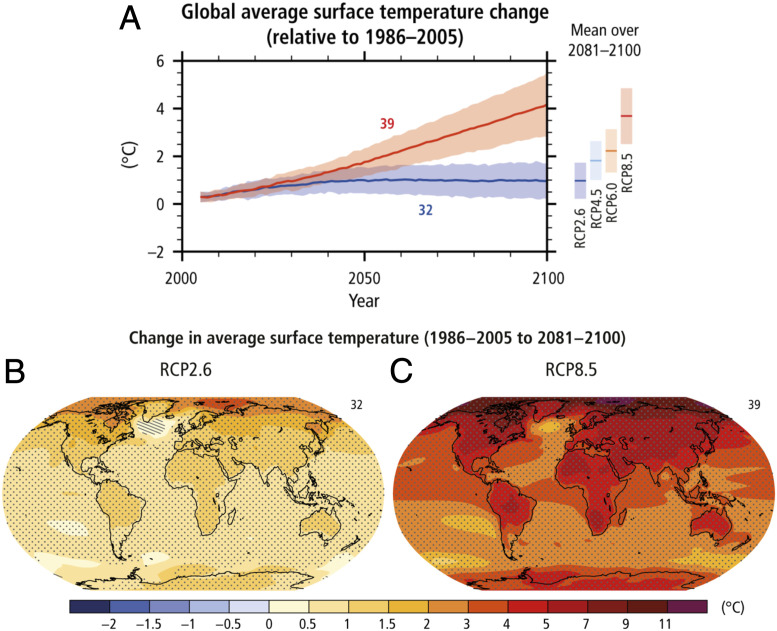
(*A*) Time series of global annual change in mean surface temperature for the period from 2006 to 2100 (relative to 1986–2005) from Coupled Model Intercomparison Project Phase 5 (CMIP5) concentration-driven experiments for scenarios RCP2.6 (blue) and RCP8.5 (red). Projections are shown for the multimodel mean (solid lines) and the 5–95% range across the distribution of individual models (shading). The number of CMIP5 models used to calculate the multimodel mean is indicated. The mean and associated uncertainties averaged over the 2081–2100 period are given for all RCP scenarios as colored vertical bars on the right-hand side. (*B* and *C*) CMIP5 multimodel mean projections for the 2081–2100 period under the RCP2.6 (*B*) and RCP8.5 (*C*) scenarios for change in annual mean surface temperature relative to 1986–2005. Adapted with permission from IPCC, 2014: Topic 2—Future Climate Changes, Risk and Impacts, in ref. 1.

Climate change and accelerated warming trigger a complex series of biological feedbacks that pose economic and social challenges for human populations. The dramatic transformation of landscapes is already observable in some regions today and is likely to accelerate in the near future. For example, in subarctic regions, species turnover rates are predicted to exceed 80% in protected areas by the end of the century (13). These transformations will affect food security and have far-reaching consequences for the physical and psychological well-being of human populations in these regions (6). Contemporary indigenous communities and small-scale subsistence farmers rely on their relations to the land and access to its natural resources for their economic and cultural reproduction. Despite their integration into capitalist modes of production and the global economy, for example, foraging activities still play an important economic role for many indigenous groups (e.g., ref. 14). Furthermore, beyond its economic importance, the land represents a locus of cultural reproduction, underpinning indigenous knowledge and memory (15, 16). Climate change poses a fundamental threat to these groups, as they themselves have eloquently pointed out (17).

For the most part, current public discourse about climate warming revolves around Western, industrialized societies despite the fact that nonindustrialized societies will likely bear the brunt of climate change (18–20). Furthermore, while maintaining biodiversity is one of the goals of climate change research, maintaining cultural diversity does not occupy the same space in public discourse. However, the loss of contemporary cultural diversity could represent an existential threat for our species. Human adaptations are the result of the dynamic relationship between cultural and biological systems. Natural selection operates on biological variation, but the archaeological record shows us that the long-term survival of our species also hinges on our ability to find cultural solutions to environmental challenges. Given the diversity of biomes currently inhabited by humans and the likelihood that they will respond differently to climate change, a range of cultural responses will be required. Cultural diversity, therefore, is the key to long-term human resilience. It is worth reflecting on the future of Western, industrialized economic/social systems and considering the possibility that other forms of social and economic organization may prove more resilient in the long run. Respecting, documenting, and conserving cultural diversity, as well as biodiversity, is therefore an essential step toward building up the resilience of human systems. Because the archaeological record captures the breadth of past human adaptations, the archaeology of climate change is well situated to highlight alternative strategies that have worked in the past and address the social and economic ramifications of global warming for a diverse global community.

## Archaeology as an Interdisciplinary Science

An archaeology of climate change emerges seamlessly from the long-standing collaboration between archaeology and the natural sciences that has provided climatological, environmental, and chronostratigraphic data critical for archaeological interpretations. Since the 19th century, this information has been used to provide a paleoenvironmental backdrop against which past human activities are studied. More recently, it, along with evolutionary theory (specifically evolutionary ecology), has provided a rich, if often implicit conceptual framework with which to study the dynamics of past human–environment interactions.

Unfortunately, archaeology and evolutionary theory have been somewhat uneasy bedfellows since the late 20th century, when the postmodernist movement fostered archaeological “post-processualism.” Postprocessualists specifically criticized archaeological research designed to explore human–environment interactions for adopting a deterministic and reductive approach. This research, they contended, assumes that external environmental processes are the principal drivers of cultural transformation while failing to acknowledge the power of historical contingency and denying human agency (21). Environmental archaeology, human evolutionary ecology, and paleoenvironmental studies in general were accused of lacking an interpretive framework capable of recognizing the importance of internal cultural processes (22, 23). Environmental approaches were also criticized for fostering a dualist approach whereby humans and their environment are treated as separate entities, a fundamentally Cartesian and Eurocentric perspective (24). As a result, while it might be relatively uncontroversial to suggest that early hominin adaptations were shaped by natural processes, the suggestion that the more complex cultural adaptations that characterize modern humans might be driven by environmental factors is far from achieving universal acceptance today (25).

The postprocessual critique was a reaction to research that inferred a causal relationship between global climate events (increasingly well-documented by the late 20th century) and contemporaneous shifts in the archaeological record without exploring the underlying mechanisms or seeking alternative explanations. The result of this critique, however, was the creation of a rift within archaeology between practitioners of evolutionary ecology and researchers experimenting with theories of agency, phenomenology, and other postmodern approaches. This rift also expressed itself in some quarters as the abandonment of broad theoretical frameworks and a retreat from generalization (26). The 21st century has seen a resurgence of interest in human–environment interactions and, with it, similar concerns about environmental determinism (27).

Methodological advances, particularly in computational ecology and archaeology, and better integration of ecological and anthropological theory, have changed the situation considerably since the 20th century, however. There are still disputes as to the relative importance of the internal and external factors that collectively drive cultural change, but as Arkush points out: “our differences lie in the extent to which we stress contingency versus process, and agency versus conditions, in the making of diverse human histories” (ref. 28, p. 200). An increasing number of scientists are striving to develop research frameworks that integrate environmental and human systems (e.g., refs. 26 and 29–31). In archaeology, integrative approaches to the study of human–environment interactions are now widely adopted, as reflected in the use of terms such as “niche construction,” “evo-devo,” “biocultural,” “socio-natural,” “socioecological,” and “ecocultural.” An emerging consensus among climate scientists also recognizes that the internal dynamics of human systems should be considered on an equal footing with the “external” natural processes with which they interact (32, 33). Interactions between human systems and the environment are seen as flowing in both directions. The archaeology of climate change capitalizes on these relatively recent developments in archaeology, in addition to developments in climate modeling (see *Climate Modeling and Environmental Reconstruction*), offering an integrative, multidisciplinary framework for identifying key aspects of climate that affect human systems (and vice versa) at different spatiotemporal scales.

## Climate Modeling and Environmental Reconstruction

Over the past few decades, methodological and theoretical advances in climate research have enabled studies of past human–environmental interactions that move beyond description and correlation to help reveal the underlying mechanisms of change in the archaeological record.

The rapid development of climate modeling since the mid-20th century led to the increased availability of paleoclimate information. The mutual benefits to be gained from working together fostered new collaborations between paleoclimate modelers, Earth scientists, and archaeologists and a revived interest in human/environment interactions. The “Stage 3 project,” for example, introduced climate modeling to a large and receptive archaeological audience while investigating the link between the pattern of human occupation in Europe during marine isotope stage 3 (MIS 3) (∼25–59 ka) and environmental conditions (34). The temporal and spatial scales of the simulated climate data produced by general circulation models (GCMs) and the degree of resolution that could be achieved were sometimes difficult to reconcile with the archaeological data, however, especially since archaeologists lack fine-grained chronological control over much of the archaeological record.

Increased computing power, the development of more complex climate models (coupling oceanic, atmospheric, and vegetation dynamics), and advances in computational archaeology have greatly improved the situation. Advances in dating techniques have increased chronological control of the archaeological record (e.g., ref. 35). Archaeologists have adopted the use of Geographic Information Systems and modeling tools, which are used to model the dynamic mechanisms underpinning human/environment interactions. Downscaling and regional modeling of simulated climate conditions have increased our ability to model human decision-making at fine spatial and temporal scales (e.g., daily foraging activities). Finally, climate researchers are increasingly aware that tracking human responses to past climate change using the paleoclimate and archaeological records is a means of assessing future climate risks and formulating a sustainable response (5, 8, 36–38).

Climate science has clearly had an impact on how research into the past is conducted. Multidisciplinary, intersectorial research teams are no longer exceptions and the many teams operating today are mature working partnerships. The benefits of these collaborations flow both ways. Climate modelers are interested in collaborating with archaeologists and other natural scientists who, in the course of their fieldwork, accumulate and date a wide range of climate and environment proxies. The pollen and faunal records, for example, make it possible to test model outcomes and adjust model design accordingly (39–42). This approach, exemplified by the Paleoclimate Modeling Intercomparison Projects (40), has yielded significant advances in the design of GCMs. Archaeologists and Earth scientists also benefit from the availability of high-resolution paleoclimate data more suitable for examining human–environment interactions over time.

At the site level, the use of carbon and oxygen stable isotopes from biogenic carbonates in sediment as well as the calibration of paleoecological data (e.g., pollen, chironomids, dinocysts, diatoms, insects, and mollusks) (43–49) based on extensive, modern training sets has permitted reliable quantitative reconstructions of environmental variables (temperature, precipitation, sea ice extent, sea level position) thought to influence human behavior and evolution through their impact on food resources, freshwater availability, habitat suitability, and other parameters (50–52). Moreover, an increased emphasis on detailed, high-resolution sampling of paleoclimatic archives, such as lake sediment cores and speleothems (where sufficient sedimentation or growth rate allow), has fostered the development of records with high temporal resolution. This has been driven, in part, by the development of analytical techniques such as X‐ray fluorescence core scanning (53) and advances in the modeling of radiocarbon and other chronological data (54), but also by research questions that focus on understanding abrupt climate change, climate transitions, and extreme events (e.g., droughts, floods) that occur over subannual to centennial timescales and are more adequate to assess short-term human responses to environmental change. At coarser spatial scales, paleoecological (e.g., Neotoma, Acer) and archaeological (e.g., Canadian Archaeological Radiocarbon Database) databases (55–57), coupled with large-scale paleoclimatic syntheses using high-quality, quantitative multiproxy datasets have facilitated regional- to continental-scale studies (58) that examine the role of climate and ecological change in driving cultural and demographic shifts, and have helped characterize climate variability, especially during the Late Glacial and Holocene at the hemispheric to global scale.

Nevertheless, high-quality, high-resolution paleoclimate records that consistently span several tens to hundreds of thousands of years are still relatively scarce. In addition, the spatial distribution of available high-resolution paleoclimatic records leaves many regions underrepresented, such as the Arctic and the tropics (59, 60). For example, in Europe many records are available for the Mediterranean domain (61), whereas the vast loess regions of northern Europe remain underexploited (but see refs. 62–64). As a consequence, data from single locations has often been used to infer past climate changes not only on regional but also on global spatial scales (65–72). Several major problems arise when dealing with a limited number of patchy proxy records. For example, the signal recorded by proxies may reflect local conditions, rather than regional or global climate changes. In addition, proxies often record seasonal changes in a given parameter, and a shift in seasonality of the recorded climate variable may lead to a flawed comparison between seasons. The development of new proxy data records from different types of archives such as lake sediments, speleothems, or loess, may require different approaches in paleoclimate reconstruction, including a wide range of micropaleontological, geochemical, or isotopic techniques (73). Multiproxy approaches are thus fundamental. While increasingly powerful computers facilitate the treatment of huge, multiproxy datasets, revisiting old datasets—sometimes the only surviving records of past climate and environmental conditions (74)—is also essential as data acquired decades ago often lack temporal resolution and would benefit from updated calibration of the proxies as well as finer isotopic and geochemical analyses now enabled with new technologies.

Another challenge related to the integration of paleoclimatic and archaeological datasets involves their spatial association. To what extent are polar ice core records, for example, representative of climate changes that would be relevant for humans living at low and mid-latitudes? Ice core records provide a detailed, long-term frame of reference for past climate conditions: They record surface air temperature at the top of the ice sheets as well as descriptions of naturally globally averaged characteristics such as greenhouse gas concentrations (e.g., ref. 75), and include indicators such as deuterium excess, related to moisture source conditions (e.g., ref. 76). However, they do not inform us about local- to regional-scale climate parameters outside the ice sheets. These are more readily documented by continental paleoenvironmental records, such as pollen, speleothems, or lake records. Archaeological sites also provide a record of regional and local climate signals that are critical for bridging global-scale paleoclimate data and archaeological datasets (e.g., refs. 11 and 77) offering detailed insights into paleoclimate change at fine spatial and temporal scales more suitable for investigating human decision making, in addition to producing controls for climate model outputs.

One of the main advantages of using proxy data associated with archaeological remains is that it reduces or eliminates chronological uncertainties between datasets. For example, pedo-sedimentary and archaeological data from Tell Leilan, Syria, allowed researchers to identify the so-called 4.2-ky event and study its human impact (78). This work has since been used to define the middle to late Holocene stratigraphic boundary (79). The last decade has also seen considerable advances in the use of oxygen isotope analyses from archaeological shell middens, which provide data on climate variability at high temporal resolution [e.g., sea surface temperature (SST) (80) and seasonality (81)]. These studies are highly relevant for documenting past environmental–human interactions because they specifically record environmental variables (e.g., SSTs, rainy season length) that directly influence human subsistence, economy, and lifeways. That being said, the spatial distribution of shell midden sites is generally restricted to marine and lacustrine shorelines, and the radiocarbon dating of shells is affected by reservoir effects, meaning that these proxies provide only a “piece of the puzzle” in the paleoclimatologist’s toolkit. In summary, paleoclimatic indicators from ice cores, marine sediment cores, and lake sediments contribute to establishing an environmental context critical for exploring questions pertaining to human evolution and adaptation.

As we have seen above, climate models are useful tools for understanding the mechanisms of past climate change. Paleoclimate simulations provide insights into how external forcings modify atmospheric and oceanic circulation, triggering past climate change. Moreover, climate models can fill the gap in paleoclimatic information between local and global scales, leading to more continuous representation of paleoclimatological conditions. Conversely, paleoclimate reconstructions offer the possibility of testing the climate model outputs for a wide variety of climate states. For example, an early to middle Holocene thermal optimum in the Northern Hemisphere (10,000–5,000 y BP) is documented in a variety of paleoclimate archives, showing a clear summer temperature warm anomaly around 6,000 y BP (60, 82, 83). However, Holocene trends in surface air temperature and SST reconstructed from proxies illustrate different regional patterns with regard to the amplitude and timing of the optimum. The North Atlantic and Norwegian Sea exhibit different patterns depending on the proxy used: SST records based on alkenones and diatoms generally show the existence of a warm early to mid-Holocene optimum, while foraminifer- and radiolarian-based temperature records show a cooling trend with warmer temperature toward the late Holocene. Using a global climate model to resolve the discordance between proxies and models (84) shows that the seasonal summer warming of the sea surface was stronger during the mid-Holocene, while the subsurface depths (<50 m) experienced a cooling. The hydrographic setting, therefore, explains the apparent contradiction between the Holocene trends exhibited by phytoplankton and zooplankton-based temperature proxy records, and the modeling work cited above shows how a climate model helps advance our understanding of the climatic changes described by these records. Progress is still needed, however, in order for us to understand decreasing trends in global temperature over the Holocene. These cannot be represented by climate models, a discrepancy Liu et al. (85) have called the “Holocene conundrum.” At a regional scale, discrepancies among proxies and between models and proxies may depend, as shown in the example above, on the distinct climate-related signal captured by each tracer as a function of the season or water depth, which also has to be taken into account (e.g., ref. 86). Furthermore, some climate reconstructions show regional differences that could be related to fine-scale features, such as ocean current properties (e.g., refs. 87–89), which would require high spatial resolution models in order to be adequately represented (e.g., refs. 90 and 91).

Climate proxy records, most of which rely on biogenic productivity driven by climate- and nonclimate-related parameters, have their own limitations and uncertainties. The predictive capability of numerical models is constrained by the sensitivity of the models to changes in forcings and boundary conditions, and to their ability to represent climate changes at adequate spatial scales, given the specificities of each climate record. Whereas both proxies and models have their respective flaws, the confrontation of proxy data and model simulations contributes to identifying critical components of the climate and environmental system, improving both approaches. Paleoenvironmental data and paleoclimate modeling are shown to be mutually beneficial, and together they enable more accurate reconstructions of past climate events at a wide range of spatial and temporal scales, and a better understanding of these recorded climate changes.

In summary, as a multidisciplinary and intersectorial community, climate scientists are now better equipped to explore the complex interactions between climate systems and human systems at multiple scales. There is also a growing awareness within funding agencies of the need to better fund research that crosses traditional disciplinary boundaries. This facilitates the development of an archaeology of climate change.

## The Archaeology of Climate Change

**Fig. 2.**
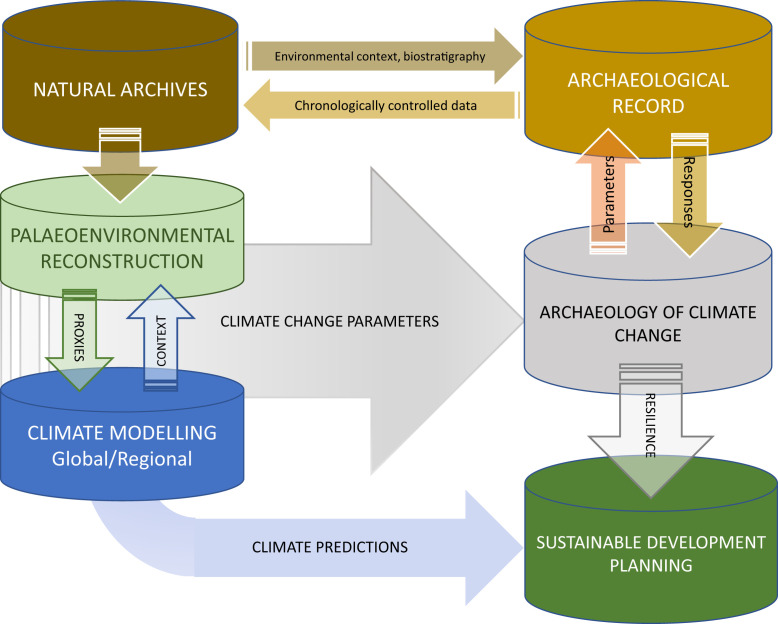
A workflow for the archaeology of climate change.

The increasing availability of high-resolution climatological and ecological reconstructions allows us to study the impact of past climate change on a human scale, one that is relevant to archaeological data and enables the reconstruction of past adaptive responses to specific types of environmental impact, including sea level change, rapid cooling and warming, climatic instability, and prolonged drought (Fig. 2). As Boivin and Crowther (92) have documented, many past adaptations to environmental change were highly successful and could be readapted to modern contexts. A comparative, cross-cultural study of the human past demonstrates that cultural diversity has been, and remains, a key element of human resilience.

The archaeology of climate change arises from the history of close collaborations between archaeologists, natural scientists, and climatologists. It builds on prior efforts to document an archaeology of environmental change (e.g., ref. 93) and harnesses 21st-century increases in computing capacity and the widespread adoption of machine-learning and modeling techniques. Computational archaeology, the use of computer-based analytical methods to study the archaeological record, is uniquely situated to leverage these developments and forms an intrinsic part of climate change research in archaeology. Early research on the impact of climate change on human systems tended to adopt an inductive approach, focusing on correlating changes in the archaeological record with climate events. Modeling approaches are used both inductively and deductively. Models can be designed to test hypotheses generated from anthropological or evolutionary theory about the sensitivity of human systems to environmental change across a range of temporal and spatial scales. They can focus on the mechanisms underlying human–environment interactions and explore their biological, social and ecological ramifications [i.e., human niche construction (94)]. Complex systems approaches provide a framework for this type of modeling, with established methods for exploring mechanistic linkages across spatial and temporal scales, how human systems react to environmental tipping points, and how patterns emerge at the population level from the collective actions of individuals (95). This includes methods for evaluating the adaptive capacity of human systems, for example by simulating the effects of changing decision rules about land use, reproduction, or mobility in response to environmental change. Cross-cultural analysis of the human past illustrates the diverse adaptive choices people have made and computational modeling allows us to further experiment with the impacts of those choices using specific high-resolution environmental reconstructions.

By way of example, the Hominin Dispersals Research Group conducts multidisciplinary research on the archaeology of climate change within an integrative, collaborative research framework. The group tests anthropologically driven research hypotheses using the archaeological record, natural archives, and climate models. Ethnographic information about the decision-making processes used by contemporary foragers, for example, was used to hypothesize that climate variability, which affects the ability to predict the distribution of resources and thus the outcome of foraging and mobility decisions, would have been a significant element of ecological risk to which past foragers will have been sensitive. High-resolution climate model outputs, developed for the project (96), were used to quantify climate variability on a subannual scale relevant for forager decision-making. Climate proxies from archaeological sites were used to test the downscaled climate model producing valuable information about the relationship between biogenic isotope signatures and climate (97). A suite of environmental variables, including climate variability, was then used to test the hypothesis using the archaeological record of Western Europe during the Last Glacial Maximum. The results of this experiment show that seasonal patterns of climate variability are key predictors of the spatial behavior of past human foragers (98). The resulting model of habitat suitability was then used to design an agent-based model to test how habitat suitability structures patterns of human land use, population structure, connectivity, and patterns of gene flow (99) with implications for cultural reproduction that are still being explored. The implications of the habitat suitability model for the adoption of different human mobility strategies were then tested using the archaeological record and lithic retouch frequencies in a diachronic study (100). The results of this collaborative research program demonstrate the value of a multidisciplinary approach for each of the disciplines involved. Climate records and climate proxies provide detailed, multiscalar information about a diversity of environmental contexts and anthropologically driven research questions result in new interpretations of the archaeological record while allowing broader hypotheses about the mechanisms underpinning human–environment dynamics, e.g., the role of stochasticity as a factor in human decision-making, to be tested.

Other sources of stochasticity linked to long-term patterns of climate change have been identified by researchers working within an archaeology of climate change framework. For example, in a context of rapid sea level and ecological change during the last deglaciation, archaeological settlement patterns show that the ancestors of today’s Cree people had a clear preference for topographically stable locations where the impact of landscape transformations linked to climate change was lessened (101). Extreme climate events, such as El Niño events, represent another potentially significant source of stochasticity; while their frequency is difficult to predict, archaeological data have been used to date and understand how farming systems in the Southern Hemisphere adapted to them (102). The frequency and scale of past climate events can now be modeled at very fine resolutions (91). The impact of an event depends on the state of the system being affected, however, which means that a cross-disciplinary approach is required in order to predict the outcome (103).

The generalization that can be drawn from this research is that human decision-making is shaped by a sensitivity to environmental stochasticity, which is one of the mechanisms through which climate change affects human populations. This generalization has potential applications for understanding the impact of climate change in contemporary situations as it suggests that stochasticity, e.g., the frequency of extreme climate events, may present a bigger challenge than rising temperatures.

The use of archaeological models to predict the impact of future climate change on contemporary societies is a relatively new concept that bridges established theories in the social and natural sciences and rests on recognition of the value of adopting a long-range perspective in climate change research. To return to our generalization with respect to stochasticity, in arctic regions where the pattern of sea ice formation is unpredictable, topographic uncertainty limits the ability to plan safe transportation and prevents people from following ancestral tracks across a landscape transformed by climate change (104, 105). This, in turn, affects both food security and the network of social interactions and relations to land that rely on human mobility and form an intrinsic part of Inuit culture. Understanding the role of topographic uncertainty in shaping human decisions could prove useful in planning sustainable development in these regions, helping to ensure the continued survival of local communities that contribute to cultural diversity on a global scale.

Humans, as a species, are thought to be uniquely adapted to dealing with climate variability (106, 107), but human groups differ in their ability to capitalize on the opportunities offered by environmental change and are not equally successful at adapting to change. The archaeological record provides evidence of a diversity of strategies adopted by different human groups in response to climate change and, more to the point, documents their outcomes. A closer look at the regional archaeological record of Southwest Asia, for example, reveals that the transition to farming was not synchronous across the region during the last Glacial/Interglacial cycle and demonstrates that a single climate event can produce very different outcomes as a result of social and geographic factors (108). In this case, Roberts et al. show that periods of favorable climate led to economic and cultural experimentation, which acted as an investment, making the society more resilient against future periods of climatic downturn.

Resilience theory, which addresses the dynamics of change in adaptive systems, has an important role to play in the archaeology of climate change. The dynamic interaction of ecological processes and historical contingency—including human action—results in irregular cycles of stability, change, and eventually transformation (109–111). The study of long-term adaptive cycles in the archaeological record has proved a fruitful avenue of research, highlighting continuities, tipping points, and loci of resilience in past socio-ecological systems, from the Pleistocene to the historical past (112–114). This approach is particularly useful for synthesizing archaeological data, contextualizing past human decision-making, and uncovering systemic relationships between natural and cultural transformations (115, 116). Because it documents complete cycles of change, instead of being limited to the study of their historical endpoints, the archaeology of climate change is uniquely positioned to contribute to resilience theory (109). Ultimately, it therefore stands to make a substantial contribution toward planning a sustainable response to global warming (9, 36, 92, 117).

Natural archives provide a record of ecosystem structure prior to large-scale anthropogenic modification, i.e., a “baseline” against which the scale of human disturbance can be measured (5). This, in turn, highlights the potential vulnerabilities and long-term sustainability of past human adaptations. Evidence for past anthropogenic disturbance in the Neotropics, for example, is best understood by considering the ecosystems with which the archaeological data interact (118). Archaeological perspectives are emerging as increasingly important in informing decision-making in the context of maximizing food security for the world’s growing population (119). The study of early farming communities offers concrete examples of the contribution archaeology can make to sustainability. Early farming communities provide models for sustainable food production, land and water management under a range of climate conditions that can be applied to contemporary situations (120). On the other hand, the fates of early farming communities, played out over the long term, also illustrate the fact that “sustainability” is a historically contingent concept (120). Archaeological models for the development of more sustainable, locally scaled adaptations to ensure food security in the coming decades include the readoption of multicropping agriculture based on the “three sisters” (i.e., corn, squash, beans) in northeastern North America (121) and strategies for mitigating risk in the event of potentially disruptive weather events such as El Niño (122). Archaeology’s contribution in this sense is twofold, in that it documents both cultigens and the tools and techniques used to cultivate and process them (123). Similar studies combining climate and human behavioral modeling with experimental farming have been conducted in the American Southwest in collaboration with Hopi maize farmers (124–126) and the long-term coevolutionary relationship between Indigenous people and food webs in Australia (127).

Thus, in addition to contributing to our understanding of sustainability, the archaeology of climate change demonstrates the role of cultural diversity as a source of human resilience. It also contributes to the protection of biodiversity (92). The importance of cultural diversity in the past also helps to highlight the role of contemporary diversity. There is growing awareness of the importance of indigenous knowledge for climate change adaptation (128) and ecosystem-based adaptation and community-based adaptation are increasingly seen as complementary approaches (19). Indigenous groups have millennia of experience and an intimate knowledge of the land that is critical to planning and enacting sustainable adaptation. Using traditional knowledge, indigenous communities manage healthy, biodiverse ecosystems, providing key services and increasing adaptive capacity (129–131). Indigenous farmers, for example, play a critical role in the maintenance of land races, which act as reservoirs of genetic diversity for a variety of food crops (132). Several ancient crops have recently been reintroduced into mainstream Western diets, contributing to the diversity of crop types included in the food chain, which is one way of ensuring the resilience of the global food supply under changing climate conditions (133). In addition, there are important ethical reasons for protecting cultural diversity that merit serious consideration (134).

## Conclusion

The archaeology of climate change is an integrated, multidisciplinary approach that incorporates resilience theory and operates within an evolutionary ecological framework. It uses the archaeological record to model human–environment interactions during past climate change events with the goal of identifying the social and ecological “tipping points” that prompt the reorganization of human systems and ecosystems at different scales and rates of change. The archaeological record also allows us to measure the relative success of past adaptative responses. Finally, it provides narrative anchors in contemporary dialogues about climate change that can be used to promote community-based adaptation. The value of adopting a long-range perspective in climate change research is absolutely essential given the scale of climate-driven global environmental transformations that are likely to occur beyond this century (4).

Much work remains to be done, climate variability is expressed in different ways across the landscape, and other sources of stochasticity, such as rates of community succession of both fauna and flora, need to be investigated more fully. More locally oriented research will be required to make climate research accessible and foster community-based responses, including active collaborations with stakeholders beyond academia (32). However, the research described above demonstrates the promise and the value of conducting an archaeology of climate change and the benefits that accrue for the participating disciplines within their own spheres of research. While it has always been notoriously difficult to fund multidisciplinary research focused on the past, the situation has been somewhat alleviated recently through the introduction of new funding streams and growing support from within climate research.

We have seen that cultural diversity, past and present, is a valuable source of resilience and climate adaptation. The archaeology of climate change has an important role to play, highlighting the importance of cultural diversity and encouraging scientists, policymakers, and stakeholders to engage with the past to help plan a sustainable future.

## Data Availability

There are no data underlying this work.
